# Injection of Fully-Defined Signal Mixtures: A Novel High-Throughput Tool to Study Neuronal Encoding and Computations

**DOI:** 10.1371/journal.pone.0109928

**Published:** 2014-10-21

**Authors:** Vladimir Ilin, Ian H. Stevenson, Maxim Volgushev

**Affiliations:** Department of Psychology, University of Connecticut, Storrs, Connecticut, United States of America; University of Antwerp, Belgium

## Abstract

Understanding of how neurons transform fluctuations of membrane potential, reflecting input activity, into spike responses, which communicate the ultimate results of single-neuron computation, is one of the central challenges for cellular and computational neuroscience. To study this transformation under controlled conditions, previous work has used a signal immersed in noise paradigm where neurons are injected with a current consisting of fluctuating noise that mimics on-going synaptic activity and a systematic signal whose transmission is studied. One limitation of this established paradigm is that it is designed to examine the encoding of only one signal under a specific, repeated condition. As a result, characterizing how encoding depends on neuronal properties, signal parameters, and the interaction of multiple inputs is cumbersome. Here we introduce a novel fully-defined signal mixture paradigm, which allows us to overcome these problems. In this paradigm, current for injection is synthetized as a sum of artificial postsynaptic currents (PSCs) resulting from the activity of a large population of model presynaptic neurons. PSCs from any presynaptic neuron(s) can be now considered as “signal”, while the sum of all other inputs is considered as “noise”. This allows us to study the encoding of a large number of different signals in a single experiment, thus dramatically increasing the throughput of data acquisition. Using this novel paradigm, we characterize the detection of excitatory and inhibitory PSCs from neuronal spike responses over a wide range of amplitudes and firing-rates. We show, that for moderately-sized neuronal populations the detectability of individual inputs is higher for excitatory than for inhibitory inputs during the 2–5 ms following PSC onset, but becomes comparable after 7–8 ms. This transient imbalance of sensitivity in favor of excitation may enhance propagation of balanced signals through neuronal networks. Finally, we discuss several open questions that this novel high-throughput paradigm may address.

## Introduction

Developing a mechanistic understanding of how neurons transform fluctuations in membrane potential, driven by synaptic inputs, into spike responses, which communicate the ultimate results of single-neuron computation, is one of the central challenges for cellular and computational neuroscience. To study this transformation under controlled conditions, a signal immersed in noise paradigm has been introduced [1], [2]. In this paradigm, a neuron is injected with a current consisting of different realizations of fluctuating noise that mimics on-going synaptic activity, and a systematic signal, such as a current step, artificial postsynaptic current (PSC), sine-wave signal, or modulation of the noise amplitude. Action potentials generated in response to repeated current injection can then provide a precise measure of the average output of the neuron in response to a specific input signal. This allows quantitative characterization of the input-output relationship during responses to defined stimuli, as they may occur in vivo. By considering a population of neurons identical to the recorded neuron this experimental paradigm for studying single neuron computation can be also used to study population encoding and computations, and inform our understanding of network function. The signal immersed in noise paradigm has been successfully applied to study signal propagation in feed-forward networks [1], [3], the speed of population responses to step-like changes of the input [2], [4], [5], [6] and characterization of the frequency transfer function of neuronal population responses [7], [8], [9], [4], [5].

One limitation of the signal immersed in noise paradigm is that it is designed to examine the encoding of only one signal under a defined condition. Studying the dependence of encoding on parameters of the signal, such as its amplitude and time course, or the properties of neurons themselves, such as firing rate, requires additional, lengthy experiments. In particular, studying the encoding of weak signals in the activity of sparsely firing neuronal ensembles – the relevant regime for cortical processing [10], [11] – requires extremely long recordings to achieve adequate statistical power [7], [4], [6]. Furthermore, although input synchrony plays important role in single neuron computation [12], [13], [14], [15], the signal immersed in noise paradigm makes it difficult to comprehensively study how *multiple* inputs may be altering spike output.

Here we introduce a novel fully-defined signal mixture paradigm, which allows us to overcome these problems and examine the effect of multiple inputs simultaneously. This paradigm exploits the fact that “noise” fluctuations of the membrane potential of a neuron in vivo represent the summed activity at its numerous synapses [16], and thus can be considered as a sum of numerous input signals. Current for injection is then synthetized as a sum of multiple artificial PSCs resulting from activity of large population of model presynaptic neurons. Because the spike timing of each presynaptic neuron is defined, we can consider PSCs from any presynaptic neuron or a combination of neurons, as “signal”, while the sum of all other inputs as “noise”, and study the encoding of a large number of different signals in a single experiment. This dramatically increases the throughput of data acquisition, allowing characterization of encoding over a broad parameter space. Here we demonstrate the applicability of this novel paradigm by characterizing the amplitude and firing-rate dependence of detection of excitatory and inhibitory PSCs from neuronal spike responses. Our results reveal that changes of the firing rate of moderate-sized neuronal populations are more sensitive to excitatory than to inhibitory inputs during the 2–5 ms following PSC onset. At later times, the sensitivity to excitatory and inhibitory inputs becomes about the same. This transient imbalance of sensitivity in favor of excitation may enhance propagation of balanced signals through neuronal networks. Finally, we discuss several groups of questions that this novel high-throughput experimental paradigm can be used to address.

## Materials and Methods

All experimental procedures used in this study were in accordance with National Institutes of Health regulations. Experimental protocols were approved by the Institutional Animal Care and Use Committee of University of Connecticut.

### Slice preparation and recording

*In vitro* intracellular recordings were made in slices of rat visual cortex. The details of slice preparation and recording were similar to those used in our previous studies [17], [4], [5], [6]. The Wistar rats (P21–P28, Charles River or Harlan, USA) were anaesthetized with isoflurane (Baxter, USA), decapitated, and the brain was rapidly removed. One hemisphere was mounted onto an agar block and 350 µm thick coronal slices containing the visual cortex were cut with a vibrotome (Leica, Germany) in ice cooled oxygenated solution. After cutting, the slices were placed into an incubator where they recovered for at least one hour at room temperature before transferring them in to the submerged-type recording chamber. The solution used during slice preparation and recording contained (in mM) 125 NaCl, 2.5 KCl, 2 CaCl_2_, 1 MgCl_2_, 1.25 NaH_2_PO_4_, 25 NaHCO_3_, 25 D-glucose and was bubbled with 95% O_2_ and 5% CO_2_. In some experiments synaptic transmission was blocked by adding 25 µM APV, 5 µM DNQX and 80 µM PTX to the extracellular solution. Chemicals were obtained from Sigma-Aldrich or Tocris.

Whole-cell recordings using patch electrodes were made from layer 2/3 pyramidal neurons, selected under visual control using Nomarski optics and infrared videomicroscopy. The patch electrodes were filled with K-gluconate based solution (in mM: 130 K-Gluconate, 20 KCl, 4 Mg-ATP, 0.3 Na_2_-GTP, 10 Na-Phosphocreatine, 10 HEPES) and had a resistance of 4–6 MΩ. Membrane potential signals recorded using Dagan BVC-700A amplifier (Dagan Corporation, USA) were low-pass filtering at 10 kHz, digitized at 20 kHz (Digidata 1440A interface and pCLAMP software, Molecular Devices) and stored in a computer for further processing. All recordings were made at 28–32°C.

### *Fluctuating current* for injection into a neuron was synthetized using two paradigms

**Figure 1:**
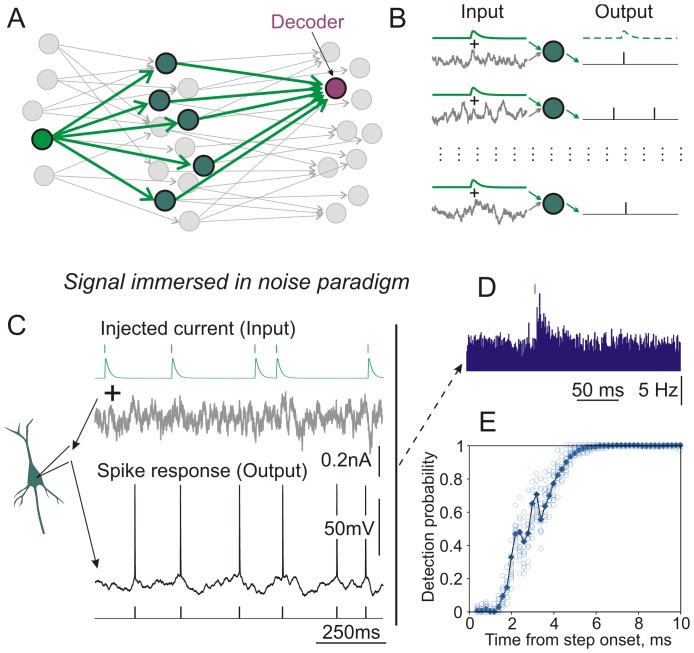
Analysis of AP encoding of a synaptic input using a *‘signal immersed in noise’* paradigm. A: A *signal immersed in noise* paradigm to study information transmission in networks of neurons. In a three-layer, feed-forward model one first-layer “source” neuron provides common input to a population of “transmitting” second-layer neurons which converge on a “decoder” neuron in the third layer. These neurons and connections are shown in green. The rest of the network is shown in gray. B: Input to neurons in the second layer consists of a “signal” – the common EPSC produced by the source neuron (green traces), and fluctuating “noise” produced by activity of other neurons (gray traces). Population firing of the transmitting neurons provides input to the decoder. C: Experimentally, population encoding in B can be mimicked by injecting current into a neuron consisting of artificial EPSCs (50 pA) immersed in fluctuating noise (SD = 110 pA). A sequence of EPSCs is shown with an enlarged Y-scale (top). Green vertical bars show onset timing of EPSCs corresponding to simulated presynaptic spikes. Timing of action potentials generated by a neuron in response to injected current was extracted from the membrane potential recording (bottom). D: Changes in a model population firing rate in response to source aEPSC. Responses to a total of N = 7223 stimuli contributed to the histogram. PSTH was obtained using aEPSC onset as a trigger for the neuron's spike response. E: Probability of detection of aEPSC from firing of N = 1000 transmitting neurons by a theoretical decoder (see scheme in A) as a function of time after the onset of the aEPSC. Filled diamond symbols: parametric detection probability. Open circles: results of bootstrapping.

In the first, *signal immersed in noise paradigm* (Fig. 1) current for injection consisted of 3 components. (i) Fluctuating current ση(t), where η(t) is an Ornstein-Uhlenbeck process with zero mean, unit variance and correlation time τ_I_ = 5 ms, and σ is the standard deviation. (ii) A signal, artificial postsynaptic potential (aPSC), synthesized as difference of two exponents, with rise time 1 ms and decay time 10 ms, and scalable peak amplitude. Timing for the aPSCs was generated by a gamma renewal process (shape k = 2, scale *θ* = 2.5), which corresponds to a rate of 5 Hz. Intervals <10 ms were resampled to avoid strongly overlapping PSCs. (iii) A DC current.

**Figure 2:**
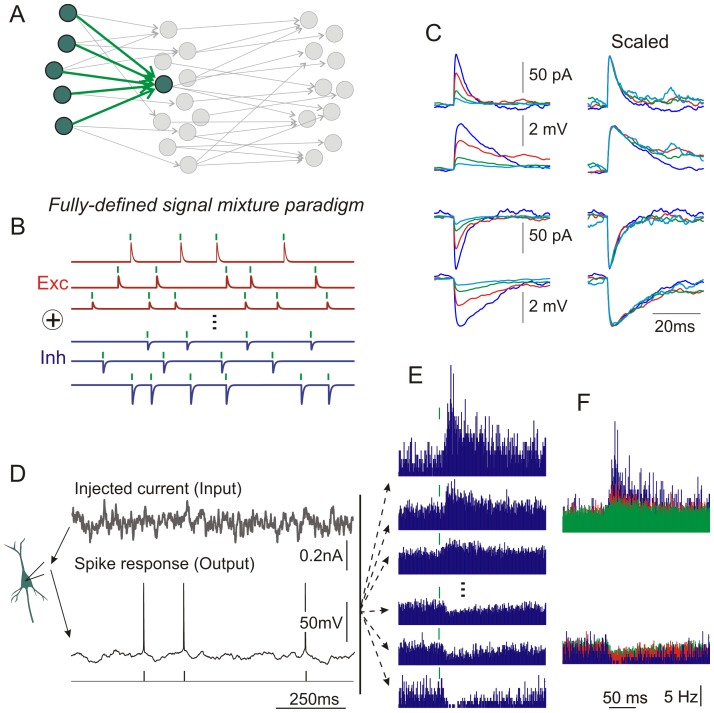
A *‘fully-defined signal mixture’ paradigm*. A: Rather than considering a transmitting neuron receiving a single input, as in the signal immersed in noise paradigm, we can also consider the three-layer network where multiple, known source signals are combined by the transmitting neuron. B: Current injected in the neuron in the fully-defined signal mixture paradigm is a sum of aEPSCs and aIPSCs of different amplitudes synthesized using the simulated activity of large number (N = 1024 in this example) of presynaptic neurons, with defined amplitudes of all aPSCs and presynaptic spike timing (green bars above each trace). C: (top to bottom) Example of averaged aEPSCs of different amplitudes, each obtained using spikes of one presynaptic neuron to trigger synthetized current for injection. Averaged membrane potential responses to these currents (aEPSPs), obtained using spikes of the same presynaptic neurons to trigger membrane potential recorded during injection of synthetized current. Traces on the right show the same responses, but amplitude-scaled. Examples of averaged aIPSCs and aIPSPs. To prevent contamination of membrane potential responses by action potentials, only fluctuating current without DC component was injected during this recording so that no spikes were generated. D: Response of a layer 2/3 pyramidal neuron to injection of a fully-defined signal mixture current. E: Changes of the population firing rate in response to aEPSCs and aIPSCs of different amplitudes. Each histogram shows changes in firing rate induced by PSPs of one particular amplitude. The spikes of individual presynaptic neurons which produced aPSCs of this amplitude were used as a trigger for postsynaptic spikes. In this way, responses to aPSCs of different amplitudes can be examined using the same recording. F: Superimposed firing rate responses from E. Note similar time course of responses to inputs of different amplitude.

In the second, *fully-defined signal mixture paradigm* (Fig. 2) fluctuating current for injection is synthetized as a summed activity of large population (N = 1024 in Fig. 2B) of model presynaptic neurons. This paradigm exploits the fact that fluctuations in the membrane potential of a neuron in vivo represent the summed activity at its numerous synapses [16]. Each presynaptic neuron contributed to the summary fluctuating current a sequence of excitatory or inhibitory aPSCs of defined amplitude. Because the timing of each aPSC is defined, we can consider trains of aPSCs resulting from activity of any presynaptic neuron or a combination of neurons, as “signal”, while the sum of all other aPSCs as “noise”, and study encoding of a large number of different signals in a single experiment (Fig. 2C). Individual aPSCs were generated as a difference of two exponentials with a rise time of 0.5 ms and decay time of 5 ms. Firing of each presynaptic neuron (mean rate 5 Hz) was simulated as gamma renewal process, and, as before, intervals <10 ms were resampled. The population of presynaptic neurons contained an equal number of excitatory and inhibitory cells and the amplitudes of excitatory and inhibitory aPSCs had a log-normal distribution, based on previous observations in paired recordings in cortical slices [18]. This resulted in a balanced fluctuating current. We note here that in real neuronal networks of the neocortex the number of excitatory synapses exceeds the number of inhibitory inputs, and the balance between excitation and inhibition is achieved through contribution of additional factors, such as higher firing rates of inhibitory neurons and differential release dynamics at excitatory and inhibitory synapses, as well as network mechanisms such as di-synaptic feed-forward inhibition or strong recurrent inhibition. However, we have opted for the straightforward case of absolute symmetry of excitatory and inhibitory inputs for several reasons. First, it is arguably the simplest way to achieve balanced input. Second, it does not introduce additional variables and does not require fine-tuning. For example, obtaining balanced currents with smaller number of inhibitory vs excitatory inputs would require higher frequency or higher amplitude at inhibitory synapses. Third, it is robust to variations of the amplitude of synaptic currents and patterns of input activity, allowing us to study broad range of these fundamental parameters. Although many input characteristics (e.g. PSC time-course, E-I ratios, E-I balance, input correlations, rates) can be studied using this fully-defined signal mixture paradigm, here we focus on a basic case where we can directly compare the sensitivity of excitatory and inhibitory inputs of different amplitudes.

In both series of experiments, the fluctuating current was scaled to produce membrane potential fluctuations of 15–20 mV amplitude, similar to membrane potential fluctuations recorded in neocortical neurons *in vivo* [16], [19], [20], [21], [22]. The SD of the scaled fluctuating current was between 70 pA and 110 pA. DC current was then adjusted to achieve a desired firing rate. All currents were injected into the soma through the whole-cell recording pipette. Current injections lasted 46 s, and were separated by a recovery period of 60–100 s.

#### Processing

Data were processed offline in Matlab (The Mathworks, Natick, MA). Spikes were detected in membrane potential traces as positive zero crossings. Spike timings were used for constructing PSTHs and for estimating PSC detection probability. Spikes generated during first 2 s after beginning of current injection were discarded from the analysis to minimize possible impact of initial spike adaptation. After the initial adaptation firing rate remained stable for the duration of current injection.

The probability of aPSC detection was quantified using a decoder [4], [6] that reports a change in the input when the constructed population firing rate falls above the 95% quantile (excitatory inputs) or below the 5% quantile (inhibitory inputs) of the pre-signal distribution. The probability of detection was estimated as a function of the time interval T (from 0.4 ms to 15 ms after the aPSC onset) for populations of N = 250, N = 500, N = 1000 and N = 2000 neurons. Single-side test statistics were computed using bootstrap analysis and from parametric (Binomial) distributions. For bootstrap analysis, we composed 100 trial sets of N (N = 250, 500, 1000, or 2000) randomly selected sweeps. For each time interval T we first used all 100 trial sets to calculate the distribution of spike counts during the pre-signal interval T and found the 5% and 95% quantile of this distribution. Next, for each trial set, we determined whether the spike count in the interval T after the PSC onset fell above the 95% quantile of the pre-signal distribution (below the 5% quantile if inhibitory). The number of trial sets, which fulfill this condition, provides an estimate of the probability for a population of N neurons to detect the PSC within time T after its onset. The whole procedure was then repeated 100 times for populations of each size (N = 250, 500, 1000 or 2000 neurons).

As an alternative to estimating detection probability with bootstrapping, parametric curves of detection probability were calculated as follows. The distribution of the number of spikes in a window of length T after the signal onset D_post_ and the distribution of the number of spikes in a window of the same length before signal onset D_pre_ were modeled as two independent binomial distributions with N equal to the number of neurons (N = 250, 500, 1000, or 2000), and estimated success probabilities P_post_ (after aPSC onset) and P_pre_ (before aPSC onset), respectively. The success probabilities P_post_ and P_pre_ were estimated using data from all recordings as average probabilities of spikes, that is
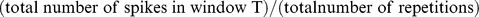
Due to the very short time windows (<10 ms) used for the analysis, any given sweep contained no more than one spike. Assuming independent sweeps with identical spike probabilities, the total number of spikes, across sweeps, can be described by a Binomial distribution. Parametric detection probabilities were then computed as the probability that a Binomial distribution B(N,P_post_) exceeds the 95% quantile (for excitatory PSCs), or is below the 5% quantile (for inhibitory PSCs) of a Binomial distribution B(N,P_pre_), that is
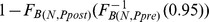
or
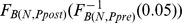
with F_B(N,Ppost)_ and F^−1^_B(N,Ppre)_ denoting the distribution and quantile function of a Binomial random variable with parameters N and P, respectively. Distributions and quantiles were computed using the Matlab programs *binoinv* and *binocdf*.

## Results

An established paradigm for studying population encoding using intracellular recording in slices is to inject in a cell a current in which a “signal”, such as an aPSC or sine-wave modulated current, is immersed in fluctuating “noise”. This *signal immersed in noise paradigm* provides a controlled setting for studying information transmission across neurons as it might occur in vivo. For instance, in a three-layer feed-forward network (Fig. 1A) each “transmitting” neuron in the second layer receives a signal from one first-layer “source” neuron, as well as numerous inputs from other neurons. These other neurons provide a background of noise which the source neuron must overcome for successful information transmission (Fig. 1B). We can assess the reliability of transmission across the population by imagining a “decoder” neuron in the third layer that receives inputs from a population of transmitting neurons. Experimentally, activity of a population of transmitting neurons is reproduced by repeatedly injecting a mixture of signal and different realizations of fluctuating noise current into a single cell (Fig. 1C). In our experiments, we scaled the amplitude of the injected current to obtain membrane potential fluctuations with an amplitude of ∼15–20 mV, and adjusted the DC current to achieve an average firing rate of ∼5 Hz. Using the spike responses of the recorded neuron to injected current, we can characterize the detectability of the source signal by the decoder receiving input from the population of transmitting neurons. The firing rate of a hypothetical population of neurons would noticeably increase after the onset of an aEPSC (Fig. 1D), and, depending on the amplitude of the signal and size of the transmitting population, a downstream decoder can detect this change in the activity of the population (N = 1000 in this example) within a few milliseconds after the aEPSC onset (Fig. 1E). Consistent with our prior observations [6], parametric estimates of detection probability using the Binomial distribution correspond closely to the estimates made with bootstrapping. This suggests that the distributions of spike counts before and after aPSC onset are well approximated by Binomial distributions.

A drawback of the signal immersed in noise paradigm is that, by design, it limits the study of neural computation to just one, or few, signals at a time (Fig. 1C,D). A typical example in Fig. 1D,E presents results from a ∼1 hour experiment during which recordings of responses to 31 episodes (46 s each) of current injection were interleaved with 60–100 s recovery intervals. Thus, it takes about 1 hour of recording to characterize the encoding of just one setting of experimental parameter values: a single amplitude for the PSC, a single output firing rate, with unchanged electrophysiological properties of the neuron. This slow rate of data acquisition, especially for low amplitude signals and/or low firing rates which require larger numbers (thousands) of signal presentations, makes complete characterization of encoding over a parameter space (e.g. including different amplitudes of excitatory and inhibitory PSCs, firing rates, control and manipulated electrophysiological properties of neurons) cumbersome.

### Fully-defined signal mixture paradigm

To overcome this drawback, we introduce a *fully-defined signal mixture paradigm*. This paradigm exploits the fact that fluctuations in the membrane potential of a neuron in vivo represent the summed activity at its numerous synapses [16]. Rather than defining “noise” based on the statistics of total input, in this paradigm we explicitly model a mixture of many inputs – in this case, from a large population of spiking presynaptic neurons. All elements of the synaptic input received by the neuron are defined: the spike timing of all simulated presynaptic neurons, as well as the amplitude and time course of simulated postsynaptic currents at each synapse (Fig. 2A). Fluctuating current for injection is synthetized as a sum of multiple signals, such as artificial excitatory and inhibitory postsynaptic currents resulting from activity of large population (N = 1024 in Fig. 2B) of model presynaptic neurons. Keeping track of the spike timings of all presynaptic neurons allows us to disentangle the effects of individual presynaptic neurons on membrane potential of the postsynaptic neuron (Fig. 2C) and and relate postsynaptic spike responses to each of the inputs (Fig. 2D,E). Figure 2E shows example histograms of postsynaptic spike responses to excitatory and inhibitory aPSCs of different amplitudes. Because the timing of each stimulus is defined, we can consider any stimulus or a combination of stimuli, as “signal”, while the sum of all other stimuli is treated as “noise”. In this way we can study the encoding of a large number of different signals in a single experiment (Fig. 2D,E). This closely mimics the situation faced by neurons in vivo, for example when neurons process sensory stimuli, relayed via specific sensory pathways, on the background of uninterrupted bombardment at all other synapses. As before, the second-layer neuron shown in green in Fig. 2A can be considered representative of all second-layer neurons, and its spiking can be used as input to a theoretical decoder (as in Fig. 1A).

**Figure 3:**
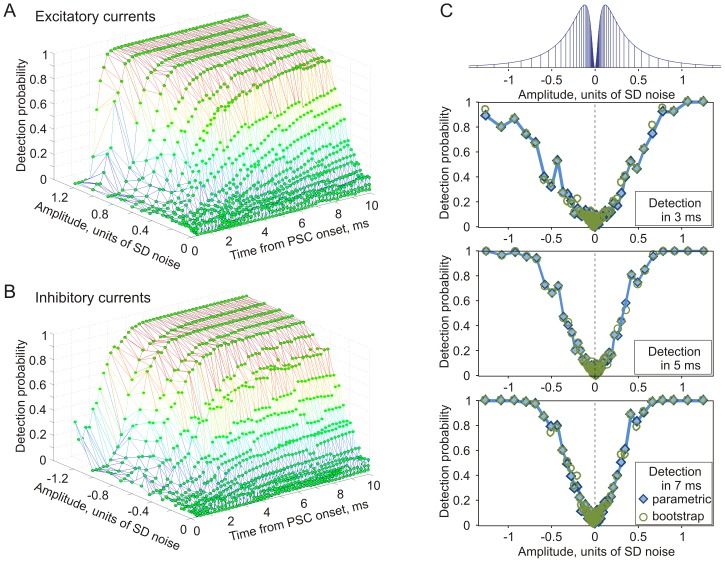
Detection probability of excitatory and inhibitory aPSCs of different amplitudes. Results from one experiment using the fully defined signal mixture paradigm. A, B: Parametric detection probability of excitatory (A) and inhibitory (B) aPSCs from population firing of N = 1000 neurons as a function of time and amplitude of the aPSC. Results were obtained in a single experiment with fully-defined signal mixture. The amplitudes are plotted in units of standard deviation (SD = 100 pA in this experiment) of the injected fluctuating current. C: Dependence of the detection probability within 3 (top), 5 (middle), and 7 ms (bottom) after aPSC onset on the amplitude of excitatory and inhibitory aPSCs. Diamond symbols denote parametric detection probability (data from A, B), while circles denote detection probability calculated from the same data using bootstrapping. Top: the distribution of PSC amplitudes used to synthetize currents for injection (as illustrated in Fig. 2B).

The fully-defined signal mixture paradigm allows us to dramatically increase the throughput of data acquisition: instead of obtaining the time-course of detection probability for one aPSC per experiment (Fig. 1), we obtain a comprehensive characterization of the time-dependence of detection probability for excitatory and inhibitory PSCs of many different amplitudes. Figure 3 presents results from one experiment using the fully-defined signal mixture paradigm. Current for injection was generated as a sum of N = 1024 presynaptic neurons, 512 excitatory and 512 inhibitory. Amplitudes of aPSCs were drawn pseudo-randomly from a discrete approximation to the log-normal distribution with μ = 0.702 and σ = 0.9355 [18], and constrained to 32 discrete, log-spaced values (Fig. 3C, top). Excitatory and inhibitory inputs had the same distribution, differing only by sign. As discussed in the Methods, we opted for this simple and robust method of achieving balanced input to simplify the parameter space and and directly compare the spike responses to excitatory vs. inhibitory inputs.

### Amplitude-dependence of PSC detection

The probability of detection for aPSCs from the population activity of N = 1000 neurons has clear dependence on both aPSC amplitude and time available for sampling population activity (Fig. 3A). Excitatory aPSC with amplitudes >∼0.5σ (σ = 100 pA in this experiment) can be detected reliably (p>0.75) very fast, within ∼3–4 ms. Smaller aPSCs required longer times for their detection, and the probability of detection of aPSCs <∼0.25σ does not reach 0.5 even after 10 ms. The probability of detection for inhibitory aPSCs (Fig. 3B) expresses similar overall patterns of dependence on PSC amplitude and time, though it takes slightly longer to detect inhibitory PSCs than excitatory PSCs of the same amplitude. The difference between probability of detection of excitatory and inhibitory PSCs is most pronounced at short intervals. In Fig. 3C, amplitude dependence of PSC detection within 3 ms is clearly asymmetric, with inhibitory PSCs detected with lower probability than excitatory PSCs of the same amplitudes. This asymmetry decreases when detection time is increased to 5 ms, and becomes negligible after 7 ms (Fig. 3C).

### Dependence of PSC detection on population size and firing rate

**Figure 4:**
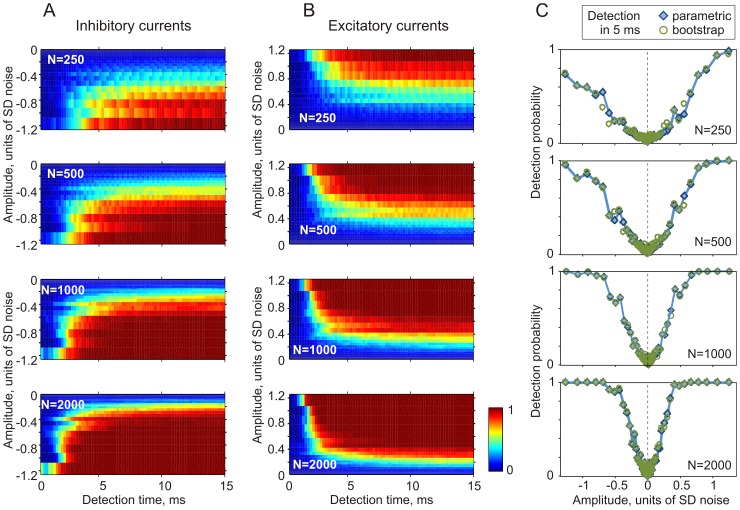
Dependence of the detection probability of excitatory and inhibitory aPSCs on the size of neuronal population. A, B: Parametric detection probability for inhibitory (A) and excitatory (B) aPSCs from population firing of N = 250, 500, 1000 and 2000 neurons as a function of time and PSC amplitude. The amplitudes are plotted in units of SD (SD = 100 pA in this experiment) of the injected fluctuating current. Detection probability (Z-axis) is color-coded. C: Detection probability within 5 ms after PSC onset as a function of PSC amplitude for populations of different sizes. Detection probability was calculated either parametrically (filled diamond symbols, data from A, B) and using bootstrapping (open circle symbols) from firing of a population of N = 250, 500, 1000 or 2000 neurons. Data from the same cell as in Fig. 3.

Two further factors had a strong influence on the probability of aPSC detection: the size of the neuronal population and the mean firing rate of the neurons. Fig. 4 shows the dependence of the probability of detection for inhibitory (Fig. 4A) and excitatory (Fig. 4B) inputs on time from aPSC onset and aPSC amplitude. Moderate-size populations of neurons (N = 250, 500) transmit excitatory PSCs into firing rate changes faster and more reliably than inhibitory PSCs (Fig. 4A,B). Note that the detection probability as a function of PSC amplitude is highly asymmetric (Fig. 4C). With the increasing size of neuronal population the latency of aPSC detection decreases, the range of reliably detectable amplitudes increases, and the difference in detection time of excitatory and inhibitory PSCs becomes negligible (Fig. 4C bottom, N = 2000).

**Figure 5:**
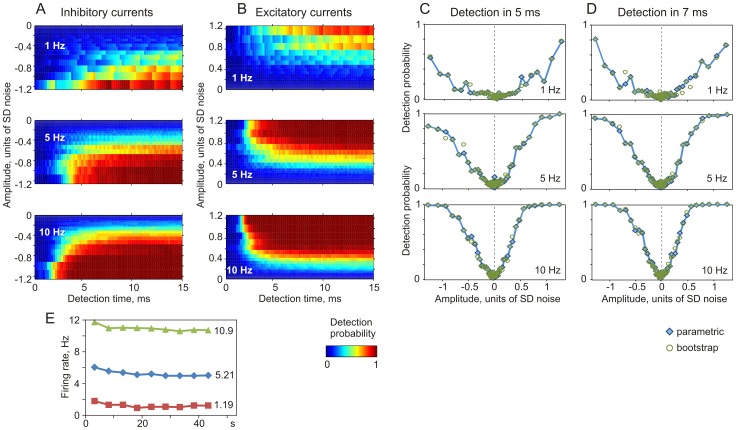
Dependence of the detection probability of inhibitory and excitatory aPSCs on postsynaptic firing rate. A, B: Parametric probability of detection for inhibitory (A) and excitatory (B) aPSCs from population firing of N = 500 neurons as a function of time and amplitude. Firing rate of neurons in the population was kept around 1 Hz (averaged 1.19 Hz), 5 Hz (5.21 Hz) or 10 Hz (10.9 Hz), as indicated. The amplitudes are plotted in units of SD (SD = 110 pA) of the injected fluctuating current. Detection probability (Z-axis) is color-coded. C, D: Dependence of the detection probability within 5 ms (C) and 7 ms (D) after onset on PSC amplitude. Detection probability calculated parametrically (filled diamond symbols, data from A, B) and using bootstrapping (open circle symbols) from activity of a populations of N = 500 neurons firing at averaged rates of 1 Hz, 5 Hz, or 10 Hz, as indicated. E: Each symbol shows firing rate during 46 s recording episodes, averaged over 5 s intervals. For each target frequency, N = 20 episodes were used; numbers on the right show gross averages.

Fig. 5 illustrates how changing the average firing rate in a population of N = 500 neurons affects PSC detectability. With low firing rate around 1 Hz (average 1.19 Hz), only the strongest PSCs can be detected reliably and the latency of their detection is long, about 5–7 ms (Fig. 5A–C, upper plots). Increasing the population firing rate to intermediate (5.21 Hz), and then to high (10.9 Hz) leads to a significant improvement in the detectability of source signals via changes in population firing (Fig. 5). These results directly demonstrate that there are important minimum firing rate constraints on information transmission by reasonably-sized (N = 500 in this example) neuronal ensembles, and that these constraints can be asymmetric for excitatory and inhibitory inputs.

Thus, the paradigm of *fully-defined signal mixture* allows us to efficiently characterize how excitatory and inhibitory PSCs are encoded in changes of the firing rates of neuronal populations. This characterization can be made within reasonable recording times in single neurons and opens up many new possibilities for comprehensive, parametric characterization of neuronal encoding. Using the signal immersed in noise paradigm we are studying input from only one presynaptic neuron, while using the fully-defined signal mixture paradigm, we are studying large number of inputs (N = 1024 in our experiments) simultaneously, which is a 1024-fold increase of the throughput of data acquisition. Data presented in Fig. 5 were obtained from a single cell to characterize spike encoding of excitatory and inhibitory PSCs with 32 different amplitudes at 3 different mean firing rates for the population of transmitting neurons (32×3 = 96 parameters). For comparison, an experiment of the same length using the signal immersed in noise paradigm (Fig. 1), would only allow us to characterize encoding with only 1 or 2 of these parameter settings.

## Discussion

Here we introduce a novel experimental paradigm for studying the transformation of input signals into spike output using fully-defined signal mixtures. This paradigm exploits the fact that “noise” fluctuations in the membrane potential of a neuron in vivo represent the summed activity at its numerous synapses [16]. Rather than modeling a signal immersed in noise, we can directly simulate synaptic input from a population of presynaptic neurons. The injected current is synthetized as a sum of multiple artificial postsynaptic currents resulting from activity of large population of model presynaptic neurons, and action potentials generated in response to injection of this current provide a precise readout of neuronal output. Because the timing of all signals contributing to the injected current, including spike timing of each presynaptic neuron is defined, we can consider any specific input or a combination of inputs as “signal”, while the sum of all other inputs as “noise”, and study encoding of a large number of different signals in a single experiment. This experimental design dramatically increases the efficiency of data acquisition, opens new possibilities for later analysis, and allows characterization of encoding over a broad parameter space.

The paradigm of fully-defined signal mixtures is a development of the established signal immersed in noise paradigm [1], [2]. In this established paradigm, current for injection is composed of a signal such as steps of current, artificial PSCs, sine-wave signals or modulation of the noise amplitude, is immersed in different realizations of fluctuating noise current that mimics on-going synaptic activity. Experimental results obtained with the use of this paradigm have led to important insights on signal propagation and encoding in feed-forward neuronal networks. They have demonstrated that synchronized activity can naturally appear when signals propagate through multilayer feed-forward neural networks [1], and that populations of cortical neurons can very quickly change their firing rate in response to abrupt changes of the input, within 1–3 milliseconds [2], [4], [5], [6]. This speed of population responses has been related to the ability of neuronal populations to phase-lock their firing to high-frequency, hundreds of Hz, periodic signals [7], [8], [9], [4], [5]. Further work has shown that the ability of neuronal populations to encode high-frequency signals depends of the activity of Kv1 potassium channels [23], and on the rapidness of the action potential onset [5]. This latter finding confirmed an earlier theoretical prediction [24], [25]. Similar approaches, injecting a signal and background noise or injecting correlated inputs, have been previously used in both experimental and theoretical analyses to assess the impact of correlated activity on neuronal firing and signal propagation in neuronal networks [1], [26], [27], [28], [29], [30]. One limitation of the signal immersed in noise paradigm, however, is that it is designed to examine encoding of one signal under one set condition. Studying the dependence of neuronal encoding and correlations on parameters of the input, e.g. amplitude and time course of postsynaptic current, or the properties of neuronal populations, e.g. firing rate and input correlations, requires additional lengthy experiments. Especially studies of encoding of weak signals in activity of sparsely firing neuronal ensembles – which is the most interesting and relevant case for cortical processing [10], [11] – require extremely long recordings [7], [4], [6].

The use of the new paradigm of fully-defined signal mixture allowed us to measure dynamic changes of population firing of cortical neurons in response to excitatory and inhibitory PSCs, with physiological time courses and amplitudes covering the broad physiological range. This analysis allowed us to identify threshold amplitudes at which excitatory and inhibitory PSCs can produce significant change of the population firing, and thus be detected in the activity of neuronal populations. The detection threshold depends on the size of neuronal population, mean firing rate, and time after the PSC onset available for analysis. Notably, even with large populations (N = 2000), high firing rates (10 Hz), and integration times of ∼7–8 ms, where detection reaches a plateau, PSCs that have amplitudes of ∼0.15σ_noise_ (corresponding to ∼15 pA PSC amplitude in our experiments) can be detected only with probability of ∼0.4. Decreasing of any of these parameters leads to a steep decrease of detection probability. These dependences are in agreement with previously reported dependence of detection of step-like input changes on the size of neuronal population and time [4], [5], and theoretically predicted rate-dependence of the sensitivity of population firing to fast input changes [24], [25], [31]. Interestingly, with decreasing population size and detection time, an asymmetry in sensitivity of population firing responses to excitatory and inhibitory inputs becomes evident. This higher sensitivity and more rapid firing rate response to excitatory inputs were predicted in earlier analysis of model neurons firing at high rates (30 or 100 Hz) [26]. Our results show that for realistic cortical neuron firing rates (∼1–10 Hz), moderate size populations (N = 250; 500), and short integration times (<5 ms), excitatory inputs are detected with higher probability than inhibitory PSCs of the same amplitude. This differential sensitivity of population firing to excitation vs. inhibition may lead to a transient, few milliseconds only, period of predominance of excitation in response to an input composed of statistically-balanced excitatory and inhibitory components. This transient imbalance may be instrumental for spreading precisely-timed waves of activity through the balanced neuronal networks [32], [33], [34].

It is important to note that the simulated presynaptic input used here was assumed to be a result of independent gamma renewal processes. Neither correlations within groups of excitatory or inhibitory synapses, nor correlations between the excitation and inhibition were implemented. In vivo presynaptic input may be substantially more coordinated [14], [35], [36], [37], and synchronous inputs can be further amplified by dendritic nonlinearities, and thus become more easily detectable [38], [39], [40]. In this scenario, very weak inputs, non-detectable alone, may still influence postsynaptic firing when synchronized. Such correlations between inputs can be easily implemented into the current for injection, making the paradigm of fully-defined signal mixture well suited for quantitative characterization of the effects of synchrony among weak inputs on information transmission through neuronal networks.

It is also important to note that in our experiments in vivo-like fluctuations of the membrane potential were induced by injection of currents with pre-defined waveforms through the somatic electrode. In contrast, membrane potential fluctuations in vivo result from changes of conductance at numerous synapses located all over the dendritic tree. The use of dynamic clamp or other real-time closed-loop experimental paradigms [41], [42] may help to circumvent the first discrepancy – induce fluctuating conductance changes instead of injection of pre-defined fluctuating current. Optogenetic approaches [43], [44], [45] may be instrumental for photo-inducing conductance changes at different parts of the dendritic tree. Combining these techniques with the fully-defined signal mixture paradigm will help to bring in vitro tools of studying neuronal encoding yet closer to conditions experienced by neurons in vivo.

### Outlook: which questions can be addressed with fully-defined signal mixture paradigm

The fully-defined signal mixture paradigm opens a number of new opportunities to study neuronal encoding and neuronal computations. First, by allowing high-throughput data acquisition and analysis of multiple inputs from the same recordings, it allows parametric characterization of encoding in different types of cortical neurons. Because data for comprehensive characterization of encoding can be obtained from individual neurons, it will allow assessment of both between-type specifics as well as within-type variability in the encoding properties of different types of neurons [46], [47], [48], [49], [50]. Second, the high-throughput of this novel paradigm, by allowing a large number of presynaptic inputs to be studied using only a few recording episodes, will also allow us to characterize the effects of postsynaptic cell properties on encoding, such as firing rate, specific ionic conductances, spike-generation mechanisms and cell membrane properties. Although varying these postsynaptic parameters does require recording of separate sets of episodes, the high-throughput paradigm will allow to acquire sufficient amount of data from the same cell, and thus make examination of the influence of postsynaptic cell properties on encoding more feasible. The properties of the input, such as correlations, rate and regularity of presynaptic firing or short-term synaptic plasticity can be systematically varied to address effects of the input structure on possible encoding schemes. Additional input signals, such as periodic sine waves of different frequencies, can also easily be added to the injected current. This will allow testing hypotheses about encoding using the power of within-subject comparisons. Third, the high-throughput of the fully-defined input paradigm makes it possible to use real neurons as “model devices” to study propagation of neuronal activity through neuronal layers and ensembles of neurons of different types. Such hybrid cell-computer experimental systems have the potential to replace simulations that fail to describe genuine electrophysiological properties and spike generation of real neurons [5], [51], [52]. Finally, modeling the sequences of action potentials generated in response to fully-defined input from a population of modeled presynaptic neurons provides a useful instrument for testing models of functional connectivity inference from large-scale recordings and verification of new tools for statistical analysis of spike trains [53], [54], [55], [56], [57]. This latter point becomes especially important in view of rapid development of electrophysiological and optical imaging techniques which are allowing simultaneous recording of spiking in increasingly large neuronal populations [58], [59].

Thus, the fully-defined signal mixture paradigm represents a novel, powerful tool to study neuronal computations performed by spike generation mechanism, but also it provides a testing instrument for development of new tools for processing large-scale spike recordings.

## References

[1] Reyes AD (2003) Synchrony-dependent propagation of firing rate in iteratively constructed networks in vitro. Nat Neurosci 6: 593–599. 12730700 10.1038/nn1056

[2] Silberberg G, Bethge M, Markram H, Pawelzik K, Tsodyks M (2004) Dynamics of population rate codes in ensembles of neocortical neurons. J Neurophysiol 91: 704–709. 14762148 10.1152/jn.00415.2003

[3] London M, Roth A, Beeren L, Haeusser M, Latham PE (2010) Sensitivity to perturbations in vivo im-plies high noise and suggests rate coding in cortex. Nature 466: 123–7. 20596024 10.1038/nature09086 PMC2898896

[4] Tchumatchenko T, Malyshev A, Wolf F, Volgushev M (2011) Ultrafast population encoding by cortical neurons. J Neurosci 31: 12171–12179. 21865460 10.1523/JNEUROSCI.2182-11.2011 PMC4225046

[5] Ilin V, Malyshev A, Wolf F, Volgushev M (2013) Fast computations in cortical ensembles require rapid initiation of action potentials. J Neurosci 33 (6) 2281–2292 10.1523/JNEUROSCI.0771-12.2013. 23392659 PMC3964617

[6] Malyshev A, Tchumatchenko T, Volgushev S, Volgushev M (2013) Energy-efficient encoding by shifting spikes in neocortical neurons. Eur J Neurosci 38: 3181–3188. 23941643 10.1111/ejn.12338 PMC3810016

[7] Koendgen H, Geisler C, Fusi S, Wang X-J, Luescher HR, Giugliano M (2008) The dynamical response properties of neocortical neurons to temporally modulated noisy inputs in vitro. Cereb Cortex 18: 2086–2097. 18263893 10.1093/cercor/bhm235 PMC3140196

[8] Boucsein C, Tetzlaff T, Meier R, Aertsen A, Naundorf B (2009) Dynamical response properties of ne-ocortical neuron ensembles: Multiplicative versus additive noise. J Neurosci 29: 1006–1010. 19176809 10.1523/JNEUROSCI.3424-08.2009 PMC6665120

[9] Higgs MH, Spain WJ (2009) Conditional bursting enhances resonant firing in neocortical layer 2–3 pyramidal neurons. J Neurosci (29) 1285–1299. 19193876 10.1523/JNEUROSCI.3728-08.2009 PMC6666063

[10] Olshausen BA, Field DJ (2004) Sparse coding of sensory inputs. Curr Opin Neurobiol 14: 481–487. 15321069 10.1016/j.conb.2004.07.007

[11] Wolfe J, Houweling AR, Brecht M (2010) Sparse and powerful cortical spikes. Curr Opin Neurobiol 20: 306–312. 20400290 10.1016/j.conb.2010.03.006

[12] Estebanez L, El Boustani S, Destexhe A, Shulz D E (2012) Correlated input reveals coexisting coding schemes in a sensory cortex. Nat Neurosci 15 (12) 1691–1699 10.1038/nn.3258 23160042

[13] Wang HP, Spencer D, Fellous JM, Sejnowski TJ (2010) Synchrony of thalamocortical inputs maximizes cortical reliability. Science 328 (5974) 106–109 10.1126/science.1183108 20360111 PMC2859205

[14] Salinas E, Sejnowski TJ (2001) Correlated neuronal activity and the flow of neural information. Nat Rev Neurosci 2 (8) 539–550 10.1038/35086012 11483997 PMC2868968

[15] Uhlhaas PJ, Pipa G, Lima B, Melloni L, Neuenschwander S, et al (2009) Neural synchrony in cortical networks: history, concept and current status. Front Integr Neurosci 3: 17 10.3389/neuro.07.017.2009 19668703 PMC2723047

[16] Destexhe A, Rudolph M, Fellous J-M, Sejnowski TJ (2001) Fluctuating synaptic conductances recreate in vivo-like activity in neocortical neurons. Neuroscience 107: 13–24. 11744242 10.1016/s0306-4522(01)00344-x PMC3320220

[17] Volgushev M, Vidyasagar TR, Chistiakova M, Yousef T, Eysel UT (2000) Membrane properties and spike generation in rat visual cortical cells during reversible cooling. J Physiol 522: 59–76. 10618152 10.1111/j.1469-7793.2000.0059m.x PMC2269736

[18] Song S, Sjöström PJ, Reigl M, Nelson S, Chklovskii DB (2005) Highly nonrandom features of synaptic connectivity in local cortical circuits. PLoS Biol 3 (3) e68. 15737062 10.1371/journal.pbio.0030068 PMC1054880

[19] Azouz R, Gray CM (2000) Dynamic spike threshold reveals a mechanism for synaptic coincidence detection in cortical neurons in vivo. Proc Natl Acad Sci U S A 97: 8110–8115. 10859358 10.1073/pnas.130200797 PMC16678

[20] Destexhe A, Rudolph M, Paré D (2003) The high-conductance state of neocortical neurons in vivo. Nat Rev Neurosci 4: 739–751. 12951566 10.1038/nrn1198

[21] Volgushev M, Pernberg J, Eysel UT (2003) Gamma-frequency fluctuations of the membrane potential and response selectivity in visual cortical neurons. Europ J Neurosci 17: 1768–1776. 10.1046/j.1460-9568.2003.02609.x 12752775

[22] Volgushev M, Chauvette S, Mukovski M, Timofeev I (2006) Precise long-range synchronization of activity and silence in neocortical neurons during slow-wave sleep. J Neurosci 26: 5665–5672. 16723523 10.1523/JNEUROSCI.0279-06.2006 PMC6675259

[23] Higgs MH, Spain WJ (2011) Kv1 channels control spike threshold dynamics and spike timing in cortical pyramidal neurones. J Physiol 589 (Pt 21) 5125–5142 10.1113/jphysiol.2011.216721 21911608 PMC3225669

[24] Fourcaud-Trocme N, Hansel D, van Vreeswijk CA, Brunel N (2003) How spike generation mecha-nisms determine the neuronal response to fluctuating inputs. J Neurosci 23: 11628–11640. 14684865 10.1523/JNEUROSCI.23-37-11628.2003 PMC6740955

[25] Naundorf B, Geisel T, Wolf F (2005) Action potential onset dynamics and the response speed of neuronal populations. J Comput Neurosci 18: 297–309. 15830166 10.1007/s10827-005-0329-8

[26] Herrmann A, Gerstner W (2001) Noise and the PSTH response to current transients: I. General theory and application to the integrate-and-fire neuron. J Comput Neuroscience 11: 135–151. 10.1023/a:1012841516004 11717530

[27] Shea-Brown E, Josić K, de la Rocha J, Doiron B (2008) Correlation and synchrony transfer in integrate-and-fire neurons: basic properties and consequences for coding. Phys Rev Lett 100: 108102. 18352234 10.1103/PhysRevLett.100.108102

[28] Ostojic S, Brunel N, Hakim V (2009) How connectivity, background activity, and synaptic properties shape the cross-correlation between spike trains. J Neuroscience 29: 10234–10253. 10.1523/JNEUROSCI.1275-09.2009 PMC6665800 19692598

[29] Tchumatchenko T, Malyshev A, Geisel T, Volgushev M, Wolf F (2010) Correlations and Synchrony in Threshold Neuron Models. Physical Review Letters 104: 058102-1-4. 20366796 10.1103/PhysRevLett.104.058102

[30] Trousdale J, Hu Y, Shea-Brown E, Josic K (2012) Impact of network structure and cellular response on spike time correlations. PLoS Comput Biology 8: e1002408. 10.1371/journal.pcbi.1002408 PMC3310711 22457608

[31] Brunel N, Chance F, Fourcaud N, Abbott LF (2001) Effects of synaptic noise and filtering on the fre-quency response of spiking neurons. Phys Rev Lett 86: 2186–2189. 11289886 10.1103/PhysRevLett.86.2186

[32] Bringuier V, Chavane F, Glaeser L, Fregnac Y (1999) Horizontal propagation of visual activity in the synaptic integration field of area 17 neurons. Science 283 (5402) 695–699. 9924031 10.1126/science.283.5402.695

[33] Okun M, Lampl I (2008) Instantaneous correlation of excitation and inhibition during ongoing and sensory-evoked activity. Nat Neurosci 11: 535–537. 18376400 10.1038/nn.2105

[34] Kremkow J, Aertsen A, Kumar A (2010) Gating of signal propagation in spiking neural networks by balanced and correlated excitation and inhibition. J Neurosci 30 (47) 15760–15768 10.1523/JNEUROSCI.3874-10.2010 21106815 PMC6633769

[35] Cohen MR, Kohn A (2011) Measuring and interpreting neuronal correlations. Nat Neurosci 14 (7) 811–819 10.1038/nn.2842 21709677 PMC3586814

[36] Singer W, Gray CM (1995) Visual feature integration and the temporal correlation hypothesis. Ann Rev Neurosci 18: 555–586. 7605074 10.1146/annurev.ne.18.030195.003011

[37] Usrey WM, Reid RC (1999) Synchronous activity in the visual system. Annu Rev Physiol 61: 435–456. 10099696 10.1146/annurev.physiol.61.1.435

[38] Margulis M, Tang CM (1998) Temporal integration can readily switch between sublinear and supralinear summation. J neurophysiology 79: 2809–2813. 10.1152/jn.1998.79.5.2809 9582247

[39] Averbeck BB, Lee D (2004) Coding and transmission of information by neural ensembles. Trends Neurosci 27 (4) 225–230 10.1016/j.tins.2004.02.006 15046882

[40] Azouz R, Gray CM (2003) Adaptive coincidence detection and dynamic gain control in visual cortical neurons in vivo. Neuron 37: 513–523. 12575957 10.1016/s0896-6273(02)01186-8

[41] Prinz AA, Abbott LA, Marder E (2004) The dynamic clamp comes of age. Trends in Neurosciences 27: 218–224. 15046881 10.1016/j.tins.2004.02.004

[42] Linaro D, Couto J, Ciugliano M (2014) Command-line cellular electrophysiology for conventional and real-time closed-loop experiments. J Neurosci Methods 230: 5–19. 24769169 10.1016/j.jneumeth.2014.04.003

[43] Boyden ES, Zhang F, Bamberg E, Nagel G, Deisseroth K (2005) Millisecond-timescale, genetically targeted optical ontrol of neural activity. Nat Neurosci 8: 1263–1268. 16116447 10.1038/nn1525

[44] Zhang F, Wang LP, Boyden ES, Deisseroth K (2006) Channelrhodopsin-2 and optical control of excitable cells. Nat Methods 3: 785–92. 16990810 10.1038/nmeth936

[45] Gunaydin LA, Yizhar O, Berndt A, Sohal VS, Deisseroth K, et al (2010) Ultrafast optogenetic control. Nature Neuroscience 13: 387–392. 20081849 10.1038/nn.2495

[46] Connors BW, Gutnick MJ (1990) Intrinsic firing patterns of diverse neocortical neurons. Trends Neurosci 13: 99–104. 1691879 10.1016/0166-2236(90)90185-d

[47] Nowak LG, Azouz R, Sanchez-Vives M V, Gray CM, McCormick DA (2003) Electrophysiological classes of cat primary visual cortical neurons in vivo as revealed by quantitative analyses. J Neurophysiol 89: 1541–1566. 12626627 10.1152/jn.00580.2002

[48] Urban N, Tripathy S (2012) Neuroscience: Circuits drive cell diversity. Nature 488: 289–290. 22895331 10.1038/488289a

[49] Druckmann S, Hill S, Schurmann F, Markram H, Segev I (2013) A hierarchical structure of cortical interneuron electrical diversity revealed by automated statistical analysis. Cereb Cortex 23 (12) 2994–3006 10.1093/cercor/bhs290 22989582

[50] Battaglia D, Karagiannis A, Gallopin T, Gutch HW, Cauli B (2013) Beyond the frontiers of neuronal types. Front Neural Circuits 7: 13 10.3389/fncir.2013.00013 23403725 PMC3566547

[51] Naundorf B, Wolf F, Volgushev M (2006) Unique properties of action potential initiation in cortical neurons. Nature 440: 1060–1063. 16625198 10.1038/nature04610

[52] Baranauskas G, Mukovskiy A, Wolf F, Volgushev M (2010) The determinants of the onset dynamics of action potentials in a computational model. Neuroscience 167 (4) 1070–1090 10.1016/j.neuroscience.2010.02.072 20211703

[53] Kaminski M, Ding M, Truccolo W A, Bressler SL (2001) Evaluating causal relations in neural systems: granger causality, directed transfer function and statistical assessment of significance. Biol Cybern 85 (2) 145–157. 11508777 10.1007/s004220000235

[54] Stevenson IH, Rebesco JM, Miller LE, Körding KP (2008) Inferring functional connections between neurons. Curr Opin Neurobiol 18: 582–588. 19081241 10.1016/j.conb.2008.11.005 PMC2706692

[55] Paninski L, Ahmadian Y, Ferreira DG, Koyama S, Rad R, et al (2010) A new look at state-space models for neural data. J Comput Neurosci 29 (1–2) 107–126 10.1007/s10827-009-0179-x 19649698 PMC3712521

[56] Chen Z, Putrino DF, Ghosh S, Barbieri R, Brown EN (2011) Statistical inference for assessing functional connectivity of neuronal ensembles with sparse spiking data. IEEE Trans Neural Syst Rehabil Eng 19 (2) 121–135 10.1109/TNSRE.2010.2086079 20937583 PMC3044782

[57] Volgushev M, Ilin V, Stevenson IH (submitted) Identifying and tracking synaptic inputs from neuronal firing: insights from in vitro experiments. 10.1371/journal.pcbi.1004167 PMC4379067 25823000

[58] Stevenson IH, Kording KP (2011) How advances in neural recording affect data analysis. Nat Neurosci 14: 139–142. 21270781 10.1038/nn.2731 PMC3410539

[59] Buzsáki G (2004) Large-scale recording of neuronal ensembles. Nat Neurosci 7: 446–451. 15114356 10.1038/nn1233

